# Protein Kinase C θ Regulates the Phenotype of Murine CD4^+^ Th17 Cells

**DOI:** 10.1371/journal.pone.0096401

**Published:** 2014-05-02

**Authors:** Katarzyna Wachowicz, Natascha Hermann-Kleiter, Marlies Meisel, Kerstin Siegmund, Nikolaus Thuille, Gottfried Baier

**Affiliations:** Translational Cell Genetics, Department of Pharmacology and Genetics, Medical University of Innsbruck, Innsbruck, Austria; University Medical Center of the Johannes Gutenberg University of Mainz, Germany

## Abstract

Protein kinase C θ (PKCθ) is involved in signaling downstream of the T cell antigen receptor (TCR) and is important for shaping effector T cell functions and inflammatory disease development. Acquisition of Th1-like effector features by Th17 cells has been linked to increased pathogenic potential. However, the molecular mechanisms underlying Th17/Th1 phenotypic instability remain largely unknown. In the current study, we address the role of PKCθ in differentiation and function of Th17 cells by using genetic knock-out mice. Implementing *in vitro* (polarizing T cell cultures) and *in vivo* (experimental autoimmune encephalomyelitis model, EAE) techniques, we demonstrated that PKCθ-deficient CD4^+^ T cells show normal Th17 marker gene expression (interleukin 17A/F, RORγt), accompanied by enhanced production of the Th1-typical markers such as interferon gamma (IFN-γ) and transcription factor T-bet. Mechanistically, this phenotype was linked to aberrantly elevated *Stat4* mRNA levels in PKCθ^−/−^ CD4^+^ T cells during the priming phase of Th17 differentiation. In contrast, transcription of the *Stat4* gene was suppressed in Th17-primed wild-type cells. This change in cellular effector phenotype was reflected *in vivo* by prolonged neurological impairment of PKCθ-deficient mice during the course of EAE. Taken together, our data provide genetic evidence that PKCθ is critical for stabilizing Th17 cell phenotype by selective suppression of the STAT4/IFN-γ/T-bet axis at the onset of differentiation.

## Introduction

To provide an effective defense against a variety of infectious agents, naïve CD4^+^ T cells differentiate into functionally diverse T helper effector subsets (Th1, Th2, Th17, iTreg and others) [1]. However, the T helper phenotype is not absolutely stable and pronounced plasticity between particular lineages exists [2]. This phenomenon is especially remarkable within the Th17 lineage [3].

Th17 cells serve to eliminate extracellular pathogens but also contribute to autoimmunity [4]. They differentiate in response to TGF-β and interleukin 6 (IL-6) [5] and produce mainly IL-17A/F and IL-22. Moreover, Th17 cells are capable of transformation into IFN-γ-producing Th1-like effectors [6] [7] [8]. This functional change depends on repetitive TCR stimulation and IL-12 or IL-23 signaling [8] [9], it increases the pathogenic potential of T cells and is required for development of proper effector responses *in vivo* [10] [11]. One of the mechanisms underlying this phenotypic plasticity might be an unstable pattern of epigenetic modifications within the *Tbx21* and *Ifng* loci in Th17 cells [12]. However, the exact molecular events regulating Th17/Th1 phenotype balance are not yet fully characterized.

Protein kinase C θ (PKCθ) is a well-known component of the immunological synapse (IS) and is essential in the signaling cascades that lead to proper NF-κB, AP-1 and NFAT activation [13]. PKCθ deficiency leads to impaired IL-2 production as well as to compromised survival and proliferation of CD4^+^ T cells [14]. Some of these defects may be overcome by other stimulating factors, such as signals from innate immunity or exogenous IL-2 [15]. Notably, PKCθ-deficient mice are able to mount relatively normal Th1, but not Th2-type immune responses [16] [17]. Due to its relevance in T cell activation and effector cell functions, PKCθ is considered as an attractive molecular drug target in inflammatory diseases [18]. Th17 cells are causative for certain autoimmune disorders, so in this context it is important to understand the exact contribution of PKCθ to the functionality of this potentially pathogenic T helper subset.

In the current study, we investigated the role of PKCθ in differentiation and function of Th17 CD4^+^ cells by using PKCθ-deficient mice [14]. While the expression of Th17 marker genes under Th17-promoting conditions (*in vivo* and *in vitro*) was unaffected by the absence of PKCθ, increased expression of the Th1-related markers, interferon gamma (IFN-γ) and T-bet, was observed. This cellular phenotypic change correlated with differences in experimental autoimmune encephalomyelitis (EAE) progression. At the molecular level, Th17 phenotypic instability could be linked to delayed *Stat4* transcriptional suppression during the early Th17 priming of PKCθ^−/−^ CD4^+^ T cells.

## Materials and Methods

### Ethics Statement

All of the mice were maintained under Specific Pathogen Free (SPF) conditions. All of the experiments complied with the Austrian Animal Welfare Law and Animal Experimental Act (BGBI. Nr.501/1988 and BGBI. Nr. 114/2012) and were approved by the Committee of the Animal Care of the Austrian Federal Ministry of Science and Research. We put efforts to minimize animals' stress and suffering by performing the immunizing injections under anesthesia and controlling animal health status regularly. At the end of experiments, animals were sacrificed by cervical dislocation.

### Mice

PKCθ*^−/−^* mice have been described previously [14]. PKCθ*^−/−^* mice were backcrossed to a 129/Sv background and used for the experiments at age of 6-12 weeks. Wild-type 129/Sv mice were used as controls.

### Experimental Autoimmune Encephalomyelitis (EAE)

EAE was induced and scored as described previously [19], with modifications. Briefly, 6-12-week-old female mice were immunized at the hind flank by injecting 250 µg of Myelin Oligodendrocyte Glycoprotein peptide (MOG_35–55_, NeoSystems, Strasbourg, France) emulsified in 100 µl of incomplete Freund's adjuvant (IFA, Thermo Fischer Scientific, Waltham, Massachusetts, USA) supplemented with 5 mg/ml Mycobacterium tuberculosis H37Ra (Difco Laboratories, Franklin Lakes, New Jersey, USA). 250 ng of pertussis Toxin (Sigma Aldrich, St. Louis, Missouri, USA) in 100 µl of PBS were injected intraperitoneally on the day of immunization and 48 h thereafter. The mice were examined daily for disease symptoms, and disease severity was graded according to the following scoring system: 0 – no symptoms; 0,5 – distal weak or spastic tail; 1 - complete limp tail; 1,5 – limp tail and hind limb weakness; 2 - unilateral partial hind limb paralysis, 2,5 – bilateral partial hind limb paralysis, 3 - complete bilateral hind limb paralysis; 3,5 – complete hind limb and unilateral partial forelimb paralysis: 4 - total paralysis or death. Mice were supposed to be immediately sacrificed when reaching the score 4. However, none of the animals reached this score during our experiments.

### Isolation of Mononuclear Cell (MNC) Infiltrates from the Central Nervous System

Mononuclear cells (MNCs) infiltrating the nervous system were isolated as described previously [19]. Briefly, brains and spinal cords were taken at onset of the disease symptoms (day 11 after immunization) and washed in PBS. Tissues were cut into pieces and digested with collagenase D (2.5 mg/ml, Roche Diagnostics, Basel, Switzerland) and DNase (1 mg/ml, Sigma Aldrich, St. Louis, Missouri, USA) at 37°C for 45 min. The digested material was passed through a 70µm cell strainer and fractionated by Percoll (GE Healthcare, Little Chalfont, United Kingdom) density gradient (70%/30%) centrifugation. MNCs were collected from interphase, washed with PBS, stained directly for FACS analysis or subjected to re-stimulation with phorbol 12,13-dibutyrate (PDBu)/ionomycin for intracellular staining of cytokine. To analyze MOG-specific T cell responses, splenocytes and lymph node cells, isolated at onset of the disease, were cultured for 3 days in the presence of MOG_35–55_ peptide (25 µg/ml) in IMDM medium supplemented with FCS and glutamine.

### *In Vitro* Cell Culture and Differentiation

Naïve CD4^+^ T cells were sorted from spleen and lymph nodes using the MACS CD4^+^CD62L^+^ T Cell Isolation Kit II (Miltenyi Biotec, Bergisch Gladbach, Germany). Cells were cultured in supplemented IMDM medium in the presence of 5 µg/ml plate-coated anti-CD3 (produced in house) and 1 µg/ml soluble anti-CD28 antibodies (BD Bioscience, San Jose, California USA).The following conditions were used: Th0 – no further cytokines or antibodies; Th17 -TGF-β [5 ng/ml], IL-6 [20 ng/ml], IL-23 [10 ng/ml], neutralizing anti-IL-4 [2 µg/ml] and anti-IFN-γ [2 µg/ml] antibodies; Th1 - IL-12 [10 ng/ml], neutralizing anti-IL-4 antibody [5 µg/ml] (eBioscience, San Diego, California, USA). Where indicated, concentration of secreted cytokines in culture supernatants was measured by Bioplex System (Bio-Rad, Hercules, California, USA), according to the manufacturer's instructions.

### Flow Cytometric Analysis

To stain for transcription factors, cells were fixed and permeabilized using Foxp3/Transcription Factor Staining Buffer Set (eBioscience, San Diego, California, USA), according to the manufacturer's instructions. For intracellular flow cytometric (ICF) staining of cytokines cells were re-stimulated for 4 h with phorbol 12,13-dibutyrate (PDBu)/ionomycin (50 ng/ml and 500 ng/ml, respectively) in the presence of Golgi Stop (BD Bioscience San Jose, California USA). Thereafter, the cells were washed, stained for surface antigens and subsequently fixed and permeabilized using the BD Cytofix/Cytoperm Kit (BD Biosciences). Data were acquired on a FACSCalibur instrument (BD Biosciences) and analyzed with FlowLogic software (eBioscience). The following antibodies were used: PE-Cy7- or FITC-conjugated anti-CD4 (RM.4–5), PE-conjugated anti-IL-17A (TC11-18H10), and APC- or PE-Cy7-conjugated anti-IFN-γ (XMG1.2) from BD Pharmingen; PE-conjugated anti-T-bet (4-B10) and APC-conjugated anti-RORγt (AFKJS-9) from eBioscience.

### Quantitative Real-Time PCR (qRT-PCR)

RNA was isolated using RNeasy Mini Kit (Qiagen, Venlo, Netherlands) according to the manufacturer's instructions. cDNA was synthesized using Omniscript Kit (Qiagen) and oligo-dT primers (Promega, Fitschburg, Wisconsin, USA) and qRT-PCR reactions were conducted using TaqMan technology (reagents were purchased from Life Technologies; Applied Biosystem, Foster City, California, USA). The reactions were run on 7500 FAST and ABI PRIM 7000 instruments (Life Technologies). TATA-Binding Protein (TBP) was used as reference gene.

### SDS-PAGE and Western Blot Analysis

Cells were harvested and resuspended in ice-cold lysis buffer (5 mM NaP_2_P, 5 mM NaF, 5 mM EDTA, 50 mM NaCl, 50 mM Tris [pH 7.3], 2% Nonidet P-40 and 50 µg/ml each of aprotinin and leupeptin). After 20 min incubation on ice, lysates were centrifuged (15,000×g, 15 min, 4°C) and supernatants were used for SDS gel electrophoresis (4–12% NuPAGE gels; NOVEX, Life Technologies, Carlsbad, California, USA). Thereafter, proteins were transferred to a PVDF membrane (Immobilion-P, Millipore, Billerica, Massachusetts, USA). The antibody-dependent chemiluminescent signal was examined by WesternBright™ Quantum reagent (Advansta, Menlo Park, California, USA) according to the manufacturer's instructions. Image acquisition was performed with Fusion FX7 instrument (Peqlab, Erlangen, Germany). Density of bands was quantified with Bio1D software (Vilber Lourmat, Eberhardzell, Germany). Following antibodies were used: primary anti-pSTAT4 (5267), anti-STAT4 (2653), anti-pSTAT3 (9131), anti-STAT3 (9139), anti-GAPDH (2118), anti-pSMAD2 (3104) and anti-SMAD2 (3103) obtained from Cell Signaling Technology (Danvers, Massachusetts, USA); anti-T-bet (H-210), anti-actin (C-11) and anti-Fyn (sc-16), anti-STAT1 (E-23) from Santa Cruz Biotechnology (Dallas, Texas, USA); anti-p-STAT1 (50-172-381) from Millipore and corresponding secondary antibodies from Thermo Fischer Scientific (Waltham, Massachusetts, USA).

### Statistical Analysis

Depending on the structure of data, obtained mean values were analyzed for statistical significance using either an unpaired Student's t-test or a grouped, one- or two-way ANOVA with the Bonferroni post hoc test. Significant differences are indicated in the graphs as follows: ^*^p<0.05, ^**^p<0.01 and ^***^p<0.001. Graphs present mean values with standard error of the mean (SEM) as error bars.

## Results

### PKCθ^−/−^ CD4^+^ T Lymphocytes Differentiate *In Vitro* into Th17 Cells with Normal Th17, but Enhanced Th1 Features

**Figure 1:**
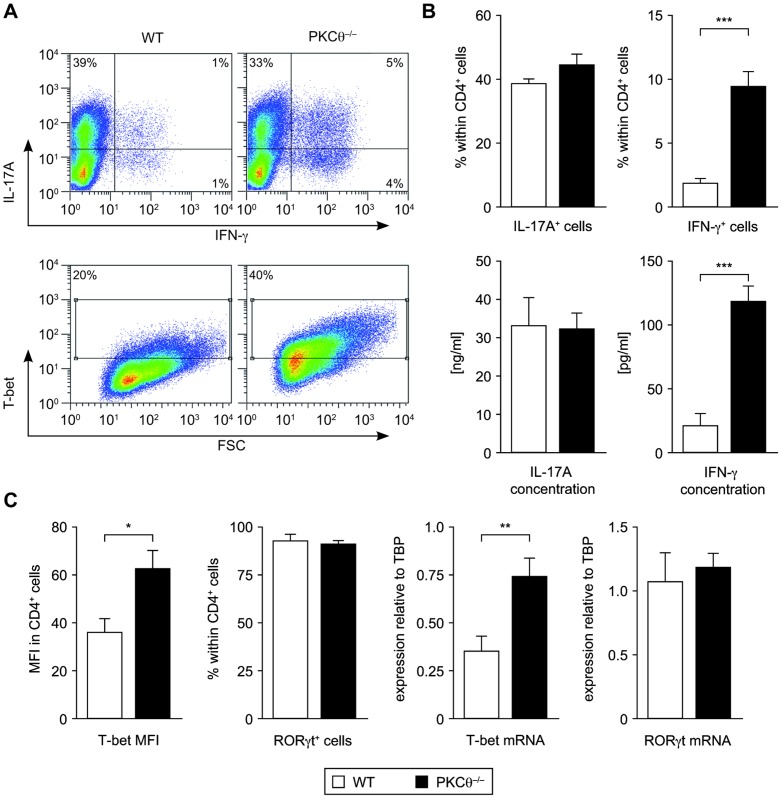
Enhanced expression of Th1-specific genes in *in vitro* differentiated PKCθ^−/−^ Th17 CD4^+^ cells. Naïve CD4^+^ T PKCθ^−/−^ and wild-type cells were cultured under Th17-polarizing condition for 4 days and analyzed for marker gene expression. A) Representative flow cytometric plots of intracellular staining of IL-17A, IFN-γ and T-bet expression. B) Quantification of flow cytometric analysis of IL-17A and IFN-γ expression (upper row) and cytokine concentrations measured in culture supernatants by Bioplex (lower row). C) Expression of the transcription factors T-bet and RORγt determined by intracellular flow cytometric staining (left side) and by quantitative real time PCR (qRT-PCR, relative expression normalized to TATA-binding protein (TBP) as a reference gene) (right side). Graphs represent combined data from at least two independent experiments, each with n = 3 per genotype; mean values with error bars indicating +/− SEM are presented. Statistical significance was assessed by a two-tailed, unpaired Student's t-test. MFI  =  mean fluorescent intensity.

To address the role of PKCθ in Th17 differentiation, naïve CD4^+^ T cells from PKCθ-proficient and -deficient mice were polarized under Th17-promoting conditions *in vitro*. The polarized Th17 cells were then analyzed for production of lineage-specific cytokines and transcription factors by flow cytometry, qRT-PCR and Bioplex System at day 4 of culture. We observed comparable expression of the major Th17 marker genes (IL-17A and RORγt) by PKCθ-deficient and wild-type (WT) CD4^+^ T cells (Fig. 1A and B). Furthermore, similar expression of other Th17-related cytokines (IL-17F, IL-21, IL-22, and TGF-β), and of transcription factor IRF4, was revealed in both genotypes (Fig. S1A). Total protein levels, as well as IL-6-induced phosphorylation of STAT3, were also unaffected (Fig. S1B). Interestingly, we detected significantly increased frequency of cells producing IFN-γ in PKCθ−/− Th17 cell cultures. In line with this, higher IFN-γ concentration was determined in the supernatants of those cultures (Fig. 1A and B). Similarly, the major Th1 transcription factor, T-bet, was increased in PKCθ−/− Th17 CD4+ cells on protein as well as on mRNA level (Fig. 1A and C). We detected severely impaired production of IL-2 by PKCθ−/− Th17 CD4+ cells, what is in accordance with previous observations showing general impairment of expression of this cytokine under PKCθ deficiency [14] (Fig. S1C). Taken together, PKCθ−/− CD4+ T cells, albeit able to develop typical Th17 features, displayed an enhanced Th1-like phenotype under Th17-polarizing conditions *in vitro*.

### PKCθ^−/−^ Th17 CD4^+^ Cells Are More Potent INF-γ Producers *In Vivo* Resulting in a Prolonged EAE-Pathogenesis

**Figure 2:**
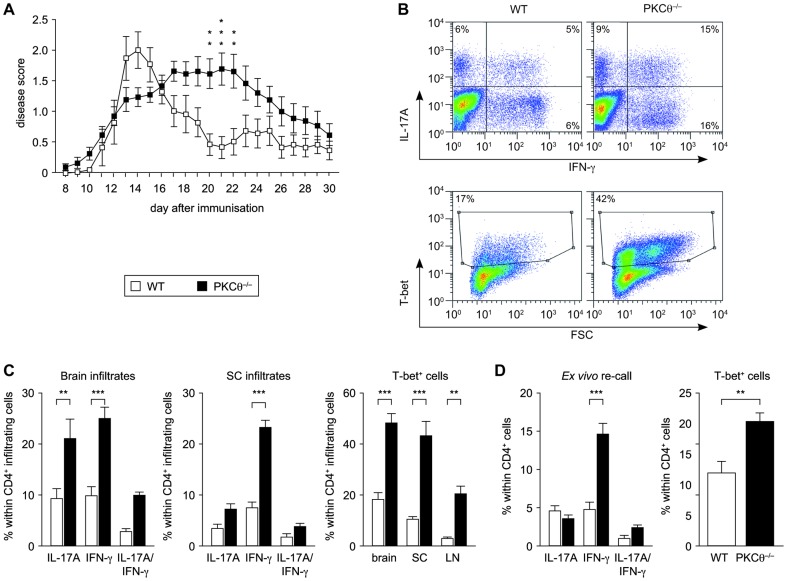
PKCθ^−/−^ CD4^+^ T cells are more potent IFN-γ producers in an autoimmune disease mouse model. A) Experimental autoimmune encephalomyelitis (EAE) disease progression in wild-type and PKCθ^−/−^ mice. The graph summarizes data from three independent experiments with total n>20 for each genotype. B) and C) Infiltrating mononuclear cells (MNC) from brain and spinal cord (SC) and lymph node (LN) cells were isolated at onset of the disease (day 11 after EAE-inducing immunisation). The cells were activated with phorbol 12,13-dibutyrate (PDBu)/ionomycin for 4 h and stained intracellulary for cytokine (IFN-γ, IL-17A) and T-bet expression. B) Representative flow cytometric plots of brain-infiltrating CD4^+^ cells. C) Quantification of cytokine-producing CD4^+^ T cell. Graphs depict results from two independent experiments with n = 3 per genotype for brain samples and from one experiment with n = 3 per genotype for SC and LNs. D) Cells from the lymph nodes and spleens were isolated at onset of the disease (day 11 after EAE-inducing immunisation). Cells were cultured *in vitro* in the presence of MOG_35–55_ peptide for 3 days, re-stimulated with PDBu/ionomycin and analyzed by intracellular flow cytometric staining for IFN-γ, IL-17A and T-bet expression. Data from one experiment with n = 12 per genotype are shown. All graphs present mean values with error bars indicating +/− SEM. Statistical significance was assessed by a two-way repeated measures ANOVA (disease progression), a two-way ANOVA with Bonferroni post hoc test (cytokines and T-bet expression in C) or by a two tailed unpaired Student's t-test (gene expression in D).

To investigate Th17 response in an *in vivo* setting, we analyzed PKCθ-deficient and wild-type mice for the development of murine experimental autoimmune encephalomyelitis (EAE), a commonly used model for multiple sclerosis (MS). After immunization with myelin oligodendrocyte glycoprotein peptide (MOG_35–55_ peptide), mice of both genotypes developed first signs of disease at day 11–12 (Fig. 2A). Furthermore, disease incidence was comparable (WT – 21 of 26, PKCθ−/− −23 of 24), and no difference in the mean maximal symptoms score was observed (WT – 2,4; PKCθ−/−−2,07; p = 0,312). However, a significant difference in the overall disease progression was apparent. Namely, the phase of severe paralysis was delayed as well as prolonged in PKCθ^−/−^ mice (starting after day 16 and lasting till ∼day 23 in PKCθ-deficient group, compared to day 12 to 15 in wild-type control). After day 25, remission phase began in animals of both genotypes.

To analyze the pathological processes on a cellular level, we isolated mononuclear cells (MNCs) infiltrating spinal cords (SC) and brains of mice at the onset of EAE symptoms (day 11). Comparable CD4^+^ T cell counts in nervous systems of wild-type and PKC-deficient animals were found (Fig. S2), so we decided to display cytokine-producing cells as a percentage within the CD4^+^ population. Increased proportion of IL-17A-producing CD4^+^ cells was found in the brains of PKCθ^−/−^ animals, but IL-17A^+^ CD4^+^ cells frequency in SC infiltrates was comparable to wild-type controls. Most strikingly, we observed more IFN-γ-producing CD4^+^ cells as well as higher frequency of T-bet^+^ CD4^+^ cells in all samples from PKCθ^−/−^ mice (Fig. 2B and C). In agreement with this, *ex vivo* MOG-specific stimulation of CD4^+^ cells isolated from lymph nodes and spleens of PKCθ^−/−^ animals suffering from EAE resulted in increased IFN-γ and T-bet frequencies, when compared to wild-type controls (Fig. 2D). Reminiscent to the *in vitro* situation (Fig. 1), wild-type and PKC-deficient CD4^+^ cells contained comparable percentage of IL-17A-producing cells (Fig. 2D). In summary, we confirmed the Th1-skewed phenotype of the PKCθ^−/−^ Th17 CD4^+^ cells *in vivo* and, furthermore, could link this to differences in the EAE disease progression between wild-type and PKCθ^−/−^ animals.

### Enhanced IFN-γ and T-Bet Expression in PKCθ^−/−^ CD4^+^ T Cells Is Limited to the Th17 Subset

**Figure 3:**
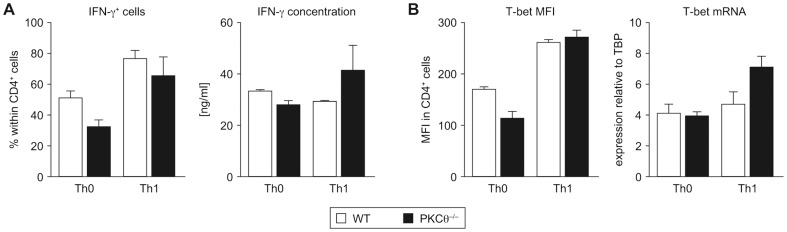
PKCθ-deficiency does not alter expression of IFN-γ and T-bet under neutral or Th1-polarizing conditions. Naïve CD4^+^ T cells were cultured under neutral (Th0) and Th1-promoting conditions for 4 days. The expression of IFN-γ (A, flow cytometric staining, Bioplex) and T-bet (B, flow cytometric staining, qRT-PCR) was analyzed. Graphs show combined data from two independent experiments, each with n = 3 per genotype; mean values with error bars indicating +/− SEM are presented. Statistical significance was assessed by a two-way ANOVA with a Bonferroni post hoc test. MFI - mean fluorescent intensity.

Considering the observed Th1-skewed phenotype of PKCθ^−/−^ Th17 cells, we determined the expression of IFN-γ and T-bet under conditions favorable to their production. When naïve CD4^+^ T cells were cultured under neutral (Th0) or Th1-promoting conditions, no significant differences in the expression of IFN-γ or T-bet (neither on protein nor on mRNA level) between wild-type and PKCθ^−/−^ T lymphocytes were observed (Fig. 3A and B).

### Early Up-Regulation of IFN-γ Production by PKCθ^−/−^ Th17 CD4^+^ Cells Is Independent of IL-23

**Figure 4:**
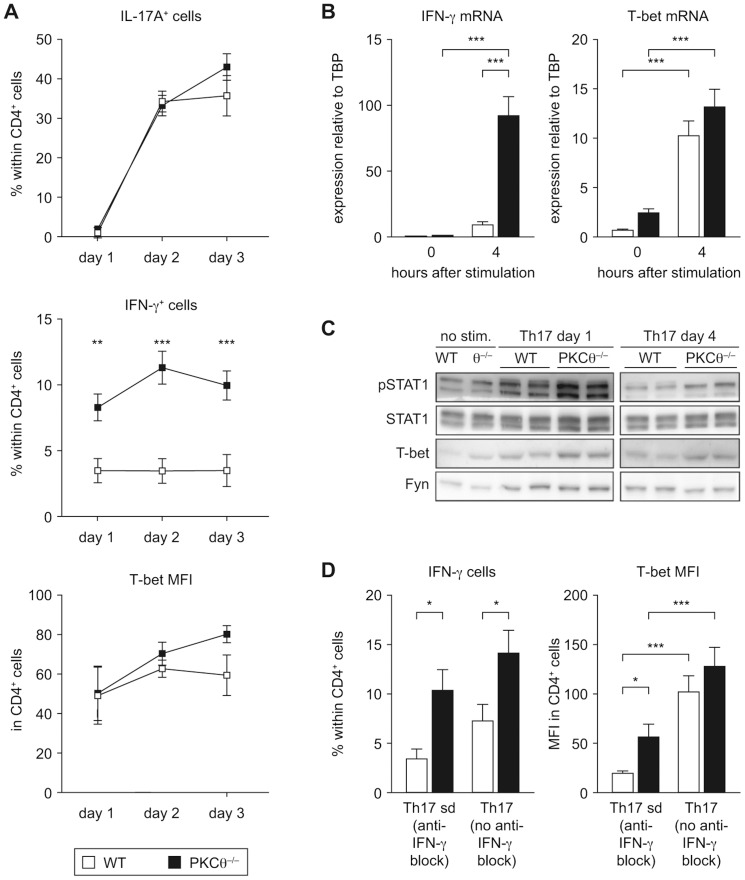
Activation of IFN-γ/STAT1/T-bet axis is enhanced in the PKCθ^−/−^ CD4^+^ T cells under Th17-polarizing conditions. Naïve CD4^+^ T cells were cultured under Th17-promoting conditions and analyzed. A) IL-17A, IFN-γ and T-bet expression was measured by intracellular flow cytometry staining on three subsequent days of Th17 differentiation. Graphs show combined data from two independent experiments, each with n = 3 per genotype. B) Early induction of IFN-γ and T-bet mRNA expression in Th17 differentiation cultures was analyzed by qRT-PCR (relative expression normalized to TBP as a reference gene). All graphs summarize data from three independent experiments, each with n = 3 per genotype. C) A representative western blot analysis of T-bet expression and total and phosphorylated STAT1 in Th17-differentiated CD4^+^ T cells (day 1 and 4). One out of three independent experiments, with two biological replicates per genotype, is shown. D) Naïve CD4^+^ T cells were cultured under Th17-promoting conditions in the presence (Th17 sd – Th17 standard conditions) or absence of anti-IFN-γ neutralizing antibodies for 4 days. IFN-γ and T-bet expression was analyzed by intracellular flow cytometric staining. Graphs show combined data from two independent experiments, each with n = 3 per genotype. All the graphs represent mean values with error bars indicating +/− SEM. Statistical significance was assessed by a two-way ANOVA with a Bonferroni post hoc test. MFI - mean fluorescent intensity.

Based on the fact that Th17 cells are able to gain the ability to produce IFN-γ [8], we decided to analyze the kinetic of IFN-γ expression during Th17 polarizing culture. In PKCθ^−/−^ Th17 cultures a larger, stable fraction of IFN-γ-secreting cells was present from as early as day 1 of differentiation, while IFN-γ-producers were scarce in wild-type cultures. However, in cultures of both genotypes, we did not detect any time-dependent increase of IFN-γ^+^ cells frequencies (Fig 4A). Similarly, IFN-γ mRNA was rapidly up-regulated only in PKCθ^−/−^ Th17-primed CD4^+^ cells (Fig. 4B). The increased expression of IFN-γ was accompanied by enhanced phosphorylation of STAT1, the major IFN-γ signaling mediator (Fig. 4C). In contrast to IFN-γ, T-bet protein and mRNA levels initially increased in the cells of both genotypes, most likely in response to the TCR stimulation [20] (Fig. 4A and B). However, following day 3 of culture, T-bet expression decreased in wild-type Th17 CD4^+^ cells, but stayed high in PKCθ^−/−^ cells (see Fig. 1A and C). This difference disappeared when no anti-IFN-γ neutralizing antibodies were added to the Th17 differentiation medium (Fig. 4D), suggesting that low secretion of IFN-γ by wild-type Th17 cells is sufficient to support T-bet expression in the absence of neutralizing anti-IFN-γ antibodies. Thus, elevated IFN-γ signaling in PKCθ^−/−^ cells may contribute to the higher T-bet protein levels observed from day 4 on [21].

**Figure 5:**
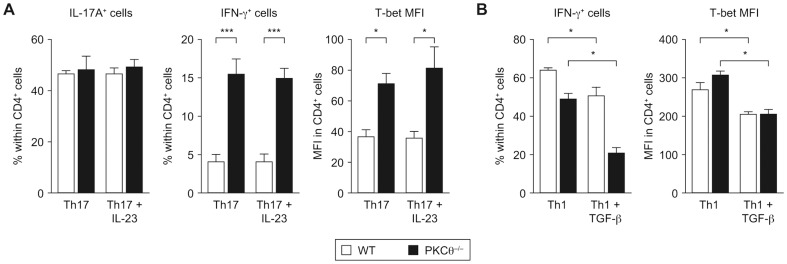
Unaltered responses to IL-23 and TGF-β in PKCθ^−/−^ CD4^+^ T cells. A) Naïve CD4^+^ T cells were cultured under Th17-promoting conditions with or without IL-23 addition for 4 days. Quantification of flow cytometric analysis of IL-17A, IFN-γ and T-bet expression is shown. Graphs present combined data from two independent experiments, each with n = 3 per genotype. B) Naïve CD4^+^ T cells were cultured under Th1-promoting conditions with and without the addition of TGF-β for 2 days. Quantification of flow cytometric analysis of IFN-γ and T-bet expression is shown. One of two independent experiments with n = 3 per genotype is presented. All graphs represent mean values and error bars indicate +/− SEM. Statistical significance was assessed by a two-way ANOVA with a Bonferroni post hoc test. MFI - mean fluorescent intensity.

IL-23 supports expansion of established Th17 cells, but it may also promote their Th1-directed phenotype transformation [7] [8]. To test whether the Th-1-skewed phenotype of PKCθ^−/−^ Th17 CD4^+^ cells may be caused by an altered sensitivity to IL-23, we conducted *in vitro* differentiation of naïve CD4^+^ T cells with and without the addition of IL-23. These experiments revealed that, as observed previously, PKCθ^−/−^ Th17 CD4^+^ cells produced IL-17A at equal frequencies as wild-type cells, but expressed more IFN-γ and T-bet, irrespective of the addition of IL-23 (Fig. 5A). Since, due to the inducible and time-delayed expression of its receptor, IL-23 acts only on differentiated Th17 cells [22], polarized Th17 cells were re-stimulated with IL-23 alone or with combination of TGF-β and IL-6. Also under these regimens, the frequencies of IFN-γ-producing cells were equally high in PKCθ-deficient, but low in wild-type cell cultures (Fig. S3A). Taken together, these data show that PKCθ^−/−^ Th17 CD4^+^ cells are able to produce increased amounts of IFN-γ constantly and independently of IL-23 receptor signaling.

### PKCθ^−/−^ CD4^+^ T Cells Remain Sensitive to TGF-β-Mediated Inhibition

Because the enhanced expression of Th1 marker genes in PKCθ^−/−^ CD4^+^ cells is limited to the Th17 subset, we considered additional Th17-specific regulatory mechanisms. TGF-β promotes Th17 differentiation in combination with IL-6 [5] and is a known suppressor of IFN-γ and T-bet [23] [24]. To test whether PKCθ deficiency alters sensitivity to inhibition by TGF-β, we cultured naïve wild-type and PKCθ^−/−^ CD4^+^ T cells under Th1-promoting conditions, with and without the addition of TGF-β. The addition of TGF-β significantly inhibited IFN-γ and T-bet expression in both genotypes (Fig. 5B). Similar results were obtained with cells kept under Th0 conditions (not shown). This is in agreement with the fact that we have observed unaltered proximal canonical TGF-β signaling in PKCθ^−/−^ CD4^+^ T cells (as measured by SMAD phosphorylation, Fig. S3B). Apart from this, we did not detect any induction of the non-canonical TGF-β signaling pathways in CD4^+^ T cells ([25] and data not shown).

### STAT4 Expression Is Differently Regulated in Wild-Type and PKCθ^−/−^ CD4^+^ T Cells during Th17 Priming

**Figure 6:**
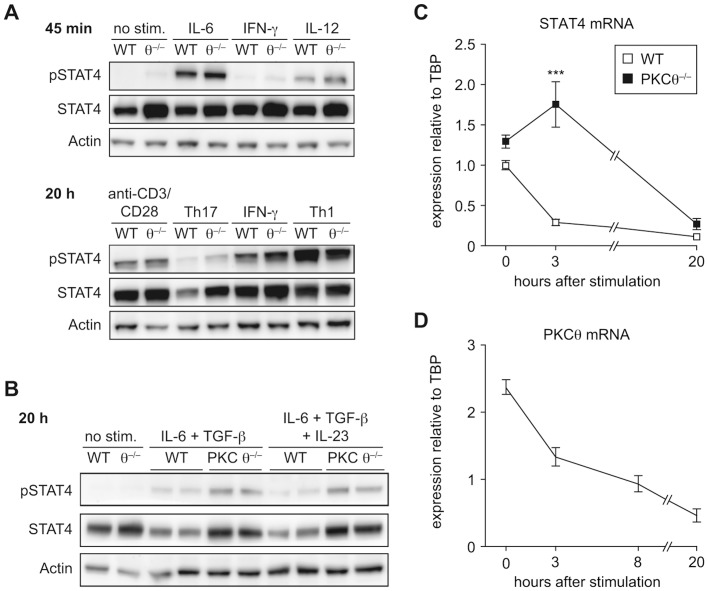
Regulation of STAT4 differs between wild-type and PKCθ^−/−^ CD4^+^ T cells during the Th17-priming. A) Western blot analysis of phosphorylated (pSTAT4) and total STAT4 protein. Naïve CD4^+^ T cell were stimulated with indicated cytokines for 45 min (upper panel) or differentiated for 20 h in the presence of anti-CD3/anti-CD28 antibodies into Th17 (IL-6/TGF-β) or Th1 (IL-12) effector cells. One representative experiment is shown. B) Western blot analysis of pSTAT4 and STAT4 in naïve CD4^+^ T cells stimulated for 20 h under Th17-promoting conditions with and without IL-23. One representative experiment, with two biological replicates of each genotype, is shown. C) *Stat4* mRNA expression was analyzed by qRT-PCR measurements (relative expression values normalized to TBP as a reference gene) during the Th17 priming of naïve CD4^+^ T cells. D) PKCθ mRNA expression was analyzed by qRT-PCR measurements (relative expression values normalized to TBP) during the Th17 priming of naïve CD4^+^ T cells. Graphs show representative data of one of three independent experiments, each with n = 3 per genotype. All graphs represent mean values and error bars indicate +/− SEM. Statistical significance was assessed by a two-way ANOVA with a Bonferroni post hoc test.

So far, our results showed that increased IFN-γ production by PKCθ^−/−^ Th17 CD4^+^ cells precedes T-bet up-regulation. Based on this observation, we focused our next analyses on factors which are known to directly promote IFN-γ expression. The most obvious candidate is STAT4, which canonically mediates IL-12-induced IFN-γ expression [20]. Thus, we determined phosphorylation of STAT4 at two different time points (45 min and 20 h) of Th17 differentiation, as well as under diverse stimulatory conditions. Short-term treatment (45 min) with IL-6 resulted in strong STAT4 phosphorylation both in wild-type and PKCθ^−/−^ CD4^+^ T cells (Fig. 6A). Importantly, this phosphorylation was transient under Th17-promoting conditions, as it was barely detectable after 20 hours of Th17-polarizing culture. Surprisingly, at this time point, PKCθ^−/−^ CD4^+^ T cells showed higher expression level of total STAT4 protein, accompanied by an increase in the amount of its phosphorylated form, than wild-type cells (Fig. 6A and B). Of note, this differential pattern of regulation was observed only under Th17-promoting, and not under neutral or Th1-promoting conditions.

Based on the protein expression kinetics (Fig. S4A), it seems that wild-type CD4^+^ T cells down-regulate STAT4 expression almost immediately after the initiation of Th17 differentiation (1–9 h). This down-regulation is much less pronounced under PKCθ deficiency. Consequently, the biggest difference in STAT4 protein levels between wild-type and PKCθ^−/−^ cells is observed after 20 hours of Th17 culture and disappears at later time points (Fig. S4A). Although IL-23 is known to promote the phosphorylation of numerous STAT proteins, including STAT4 [26], the presence of this cytokine had no effect on the STAT4 dynamics in our experiments (Fig. 6B).

To determine which factors in the Th17-differentiation medium were responsible for STAT4 down-regulation, we activated naïve CD4^+^ T cells with anti-CD3/anti-CD28 antibodies and cultured them in the presence of IL-6 and TGF-β, separately or in combination. After 20 hours, we observed that TGF-β was required to induce STAT4 down-regulation, but the differences between the genotypes were visible only in the presence of both cytokines (Fig. S4B).

Since STAT4 protein expression can be regulated by both transcriptional regulation [27] [28] and protein degradation [29], we wanted to investigate which of these processes contributes to the differences observed between wild-type and PKCθ^−/−^ CD4^+^ T cells during Th17 priming. PKCθ is known to be involved in the regulation of numerous transcription factors [13], therefore, we first investigated *Stat4* mRNA levels within the initial hours of Th17 polarization. *Stat4* mRNA was rapidly down-regulated in wild-type CD4^+^ T cells, while this process was delayed in the absence of PKCθ (Fig. 6C). These differences in mRNA kinetic corresponded to higher STAT4 protein levels in PKCθ^−/−^ Th17 CD4^+^ cells. Of note, also PKCθ mRNA expression was found to be down-regulated early during Th17 differentiation (Fig. 6D). This phenomenon, already described in the literature [30], was observed by us also under different stimulation conditions (not shown). This observation goes in line with the assumption that PKCθ works as a regulatory factor during the early phase of phenotype determination and its activity declines once the effective *Stat4* suppression is established.

## Discussion

The current study identified a new and entirely unexpected role of PKCθ in regulating the phenotype of Th17 cells subset. *In vivo* and *in vitro* derived PKCθ^−/−^ Th17 CD4^+^ cells, although able to express normal amounts of subset-specific genes, are phenotypically unstable and show a strong tendency to gain Th1-like features. This may affect their physiologic functionality, as well as pathogenic potential.

Our results are in contrast to previous investigations, which have suggested a permissive role of PKCθ in the development of Th17 responses and autoimmunity [31] [32] [33]. According to these authors, PKCθ is required for effective IL-17 expression, STAT3 activation and susceptibility to EAE development. Of note, we reproducibly did not observe any of those phenomena. It should be underlined, however, that the cited results were obtained with a transgenic mouse strain generated with a different knockout strategy than the one used in our laboratory [34]. Distinct phenotypic and biochemical differences between these two knockout models have been previously reported [14]. Moreover, the different genetic background of the mice (C57BL/6 versus 129/sv used in our study) may also contribute to the observed discrepancy. In a couple of disease models, 129/Sv mice show responses different from the C57BL/6 strain [35] [36]. For example, the 129/Sv strain is described to be less susceptible to EAE induction and to mount stronger IFN-γ responses against pathogenic insults [37]. Supporting this hypothesis, one group reported previously that the effect of PKCθ deficiency was dependent on the genetic background of the mouse strain [38]. Above that, it should to be taken into account that many effects of PKCθ deficiency might be overcome by additional stimulation or partially compensated by redundant PKC isoforms [15] [39].

General progression of autoimmune conditions is regulated by the counter-play between different Th subsets and cytokines. In example, the pathogenesis of EAE is driven by both Th17 and Th1 mechanisms, and changes in the balance of these two T helper subsets result in alterations in the disease progression [40]. IFN-γ has been shown to play a protective role in acute inflammatory processes, especially within the nervous system [41], and the genetic ablation of IFN-γ signaling in mice leads to an exacerbated form of EAE [42]. On the other hand, the same cytokine plays a role in chronic and late inflammatory responses [6] [7]. In addition to the requirement of T-bet for Th17 cells-induced encephalitogenicity [43], elevated IFN-γ production in PKCθ-deficient mice may ameliorate disease severity during acute stages after immunization, but also may promote an elongation of sever disease phase, as observed in our experimental system.

Here, we examined the molecular basis of phenotypic instability of PKCθ^−/−^ Th17 CD4^+^ cells. We conclude that the observed Th1-directed skewing of the phenotype is not due to the “late developmental plasticity”. Instead, it might be a consequence of altered cellular signaling during an early Th17-priming phase, which then results in an incomplete suppression of Th1-typical genes. This hypothesis is based on the increased IFN-γ expression observed at the beginning of Th17 polarization. Although restricted to the Th17 subset, the enhanced expression of IFN-γ by PKCθ^−/−^ CD4^+^ cells was not caused by an altered TGF-β responsiveness. We propose that PKCθ-dependent regulation of STAT4 influences IFN-γ production under Th17-promoting conditions. STAT4 is most widely known as an IL-12-activated Th1 inducer [20]. Moreover, it plays also more subtle roles in determination and function of other T helper subsets; for instance, STAT4 has been shown to control the late IL-23-dependent expression of IFN-γ in Th17 cells [44]. Similarly, presence of STAT4 and T-bet is required to permit plasticity of memory CD4^+^ T cells - namely, to enable IFN-γ expression in Th2-differentiated lymphocytes [45]. We observed prominent differences between STAT4 regulation in PKCθ^−/−^ and wild-type CD4^+^ T cells in the presence of Th17-promoting cytokines. Both total protein and phosphorylation levels of STAT4 were higher in PKCθ^−/−^ CD4^+^ T cells during the Th17 priming, what corresponds with the observed enhancement of IFN-γ expression.

The protein level of STAT4 is the combined consequence of active transcription [29] and protein degradation, the later being mainly induced by a strong and persistent stimulation [46]. Since numerous reports have postulated that transcriptional suppression is the main mechanism of STAT4 down-regulation [27] [28] [47], we focused on investigating *Stat4* mRNA transcripts. The obtained data correlated well with the observed changes in STAT4 protein levels. As STAT4 mainly influences transcription by providing permissive chromatin modifications [48] [49], its prolonged presence in cells allows continuous IFN-γ production. How exactly PKCθ influences STAT4 levels remains unknown. However, we postulate that PKCθ is involved early during the Th17-priming phase (up to 24 h). This time frame is in agreement with the fact that PKCθ is activated immediately after TCR engagement. Moreover, we observed a strong down-regulation of PKCθ expression after the Th17 priming, mechanistically explaining its selective role only during this early phase of differentiation. PKCθ positively regulates a wide range of transcription factors from the AP-1, NF-κB and NFAT families. Most of these act as transcription activators, but some may have inhibitory properties [50] [51]. However, the regulation of STAT4 by PKCθ may also be less direct. One report from human cells showed that PKCθ influences the transcription of miRNA clusters [52] and, in turn, controls expression of many other genes. This type of complex regulation may serve as a fine-tuning of a cellular phenotype.

In conclusion, our data are the first to provide experimental evidence that PKCθ is an early factor involved in developing a stable Th17 cell phenotype. Mechanistically, it acts as a reprogramming element that suppresses the STAT4/IFN-γ/T-bet axis. Thus, PKCθ is an essential link between TCR signaling and inhibition of Th1-typical genes during Th17 immunity. STAT4, T-bet and IFN-γ are often implicated in autoimmune and inflammatory disorders [41] [43] [53]. Consequently, a new aspect of Th17/Th1 phenotype regulation described here has to be carefully considered in PKCθ-targeted immunosuppression therapy regimens.

## Supporting Information

Figure S1 **Th17-related genes expression and IL-2 production defect in** ***in vitro*** **differentiated PKCθ^−/−^ Th17 CD4^+^.** A) Naïve CD4^+^ T cells were cultured under Th17-promoting conditions for 4 days and then expression of subset-characteristic genes was measured by qRT-PCR (relative expression normalized to TATA-binding protein (TBP) as a reference gene) Graphs represent combined data from two independent experiments, each with n = 3 per genotype. B) STAT3 phosphorylation (pSTAT3) and total protein levels (STAT3) after stimulation of freshly isolated CD4^+^ T cells. Stimulation conditions are indicated in the figure. This is one representative experiment out of 3. C) IL-2 concentration measured by Bioplex System in supernatants of cell cultures kept under neutral (Th0) or Th17-polarizing conditions for 3 days. Graphs present results from two independent experiments for Th17 samples and from one experiment for Th0 conditions; in each experiment n = 3 per genotype. All graphs represent mean values and error bars indicate +/− SEM. Statistical significance was assessed by a two-tailed unpaired Student's t-test.
(TIF)

Figure S2 **Cells numbers and CD4^+^ cells frequencies within MNCs infiltrating central nervous system.** Infiltrating mononuclear cells (MNCs) were isolated from brains and spinal cords (SC) of mice at the onset of disease symptoms (day 11 after EAE-inducing immunisation). A) Total numbers of MNCs infiltrating brain and SC. B) CD4^+^ cells fractions within the infiltrating MNCs. Graphs show combined data from two independent experiments (brain) or from one experiment (SC), each with n = 3 per genotype. All graphs represent mean values and error bars indicate +/− SEM. Statistical significance was assessed by a two-tailed unpaired Student's t-test.
(TIF)

Figure S3 **Unaltered responses to IL-23 and TGF-β in PKCθ^−/−^ CD4^+^ T cells.** A) Naïve CD4^+^ T cells were differentiated under Th17-promoting conditions for 4 days and then re-stimulated in the presence of anti-CD3/CD28 antibodies, with IL-23 or with a combination of IL-6/TGF-β. After 3 days of re-stimulation, the cells were analyzed by intracellular flow cytometric staining for IFN-γ and T-bet expression. Graphs represent combined data of two independent experiments, each with n = 3 per genotype. B) Equal SMAD2/3 phosphorylation and protein levels in freshly isolated WT and PKCθ^−/−^ CD4^+^ cells treated for 30 min with 5 ng/ml of TGF-β or unstimulated; quantification of the western blot acquisitions. Graphs show combined data from two independent experiments with total n = 3 per each genotype and condition. All graphs represent mean values and error bars indicate +/− SEM. Statistical significance was assessed by a 2-way ANOVA with a Bonferroni post hoc test.
(TIF)

Figure S4 **STAT4 regulation during Th17 polarization in WT and PKCθ^−/−^ CD4^+^ T cells.** Naïve WT and PKCθ^−/−^ CD4^+^ T cells were stimulated by anti-CD3/CD28 antibodies for the indicated times and with the addition of indicated combinations of cytokines. A) Changes of STAT4 phosphorylation and total protein level during Th17 polarization. B) STAT4 regulation in response to different stimulation conditions in the priming phase of Th17 differentiation. Western blots of the representative experiments are shown.
(TIF)

File S1 **ARRIVE Guidelines Check list.** Filled checklist of ARRIVE (Animal Research: Reporting of In Vivo Experiments) guidelines concerning conducting of animal experimentation in scientific work.
(DOCX)
