# Supplementary figures and images for: Protein Kinase C θ Regulates the Phenotype of Murine CD4+ Th17 Cells
Source: PLoS One. 2014 May 2;9(5):e96401. doi: 10.1371/journal.pone.0096401 (PMC4008503; doi:10.1371/journal.pone.0096401)

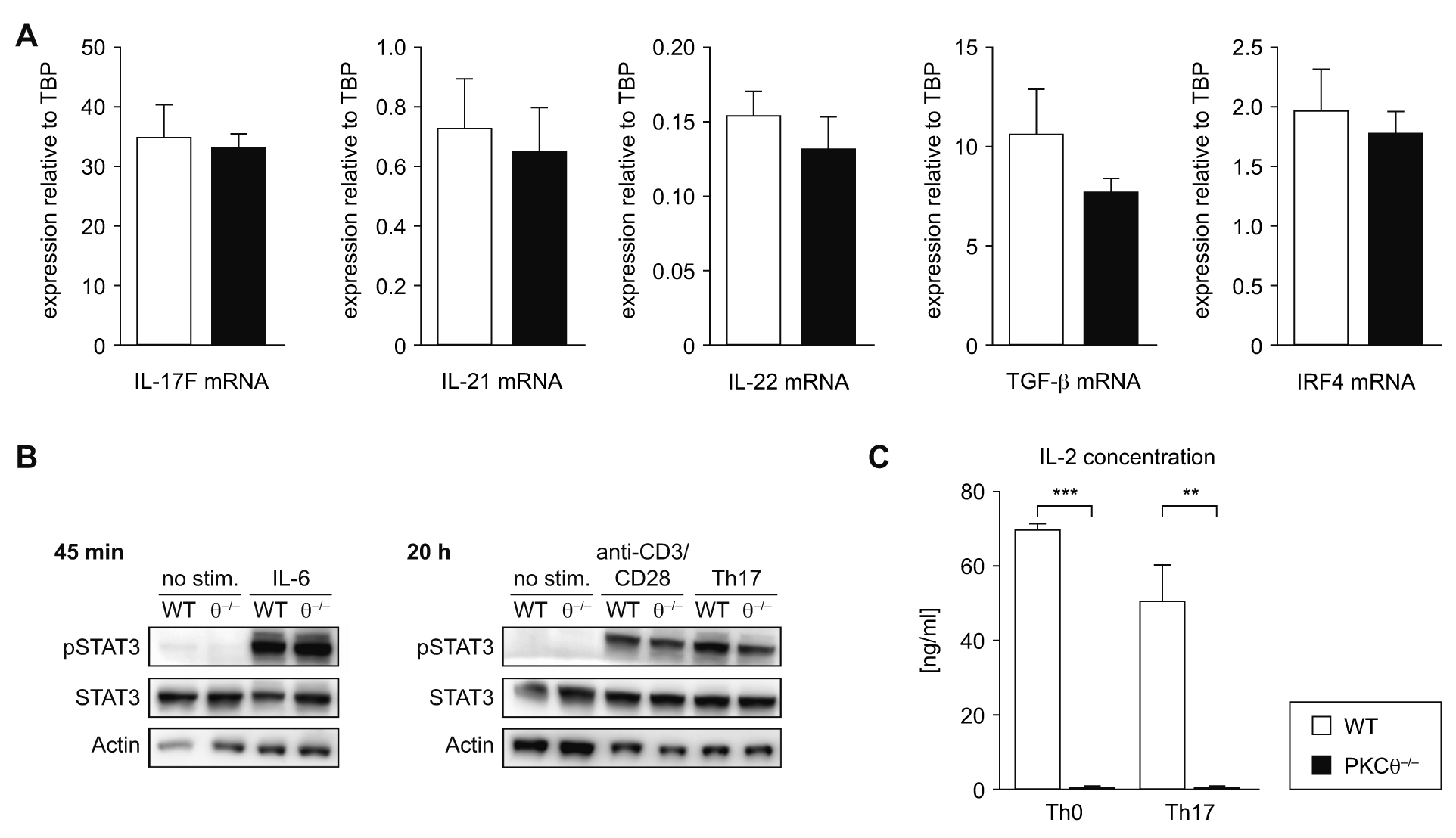

Supplement: Figure S1 — Th17-related genes expression and IL-2 production defect in in vitro differentiated PKCθ−/− Th17 CD4+. A) Naïve CD4+ T cells were cultured under Th17-promoting conditions for 4 days and then expression of subset-characteristic genes was measured by qRT-PCR (relative expression normalized to TATA-binding protein (TBP) as a reference gene) Graphs represent combined data from two independent experiments, each with n = 3 per genotype. B) STAT3 phosphorylation (pSTAT3) and total protein levels (STAT3) after stimulation of freshly isolated CD4+ T cells. Stimulation conditions are indicated in the figure. This is one representative experiment out of 3. C) IL-2 concentration measured by Bioplex System in supernatants of cell cultures kept under neutral (Th0) or Th17-polarizing conditions for 3 days. Graphs present results from two independent experiments for Th17 samples and from one experiment for Th0 conditions; in each experiment n = 3 per genotype. All graphs represent mean values and error bars indicate +/− SEM. Statistical significance was assessed by a two-tailed unpaired Student's t-test. (TIF) [file pone.0096401.s001.tif]

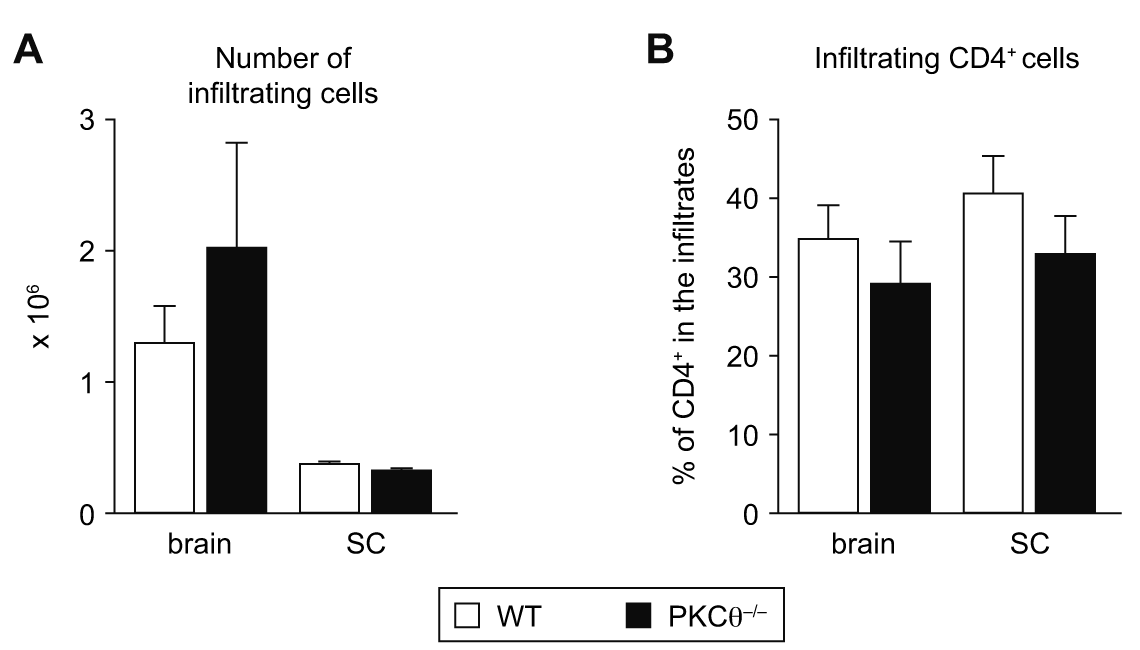

Supplement: Figure S2 — Cells numbers and CD4+ cells frequencies within MNCs infiltrating central nervous system. Infiltrating mononuclear cells (MNCs) were isolated from brains and spinal cords (SC) of mice at the onset of disease symptoms (day 11 after EAE-inducing immunisation). A) Total numbers of MNCs infiltrating brain and SC. B) CD4+ cells fractions within the infiltrating MNCs. Graphs show combined data from two independent experiments (brain) or from one experiment (SC), each with n = 3 per genotype. All graphs represent mean values and error bars indicate +/− SEM. Statistical significance was assessed by a two-tailed unpaired Student's t-test. (TIF) [file pone.0096401.s002.tif]

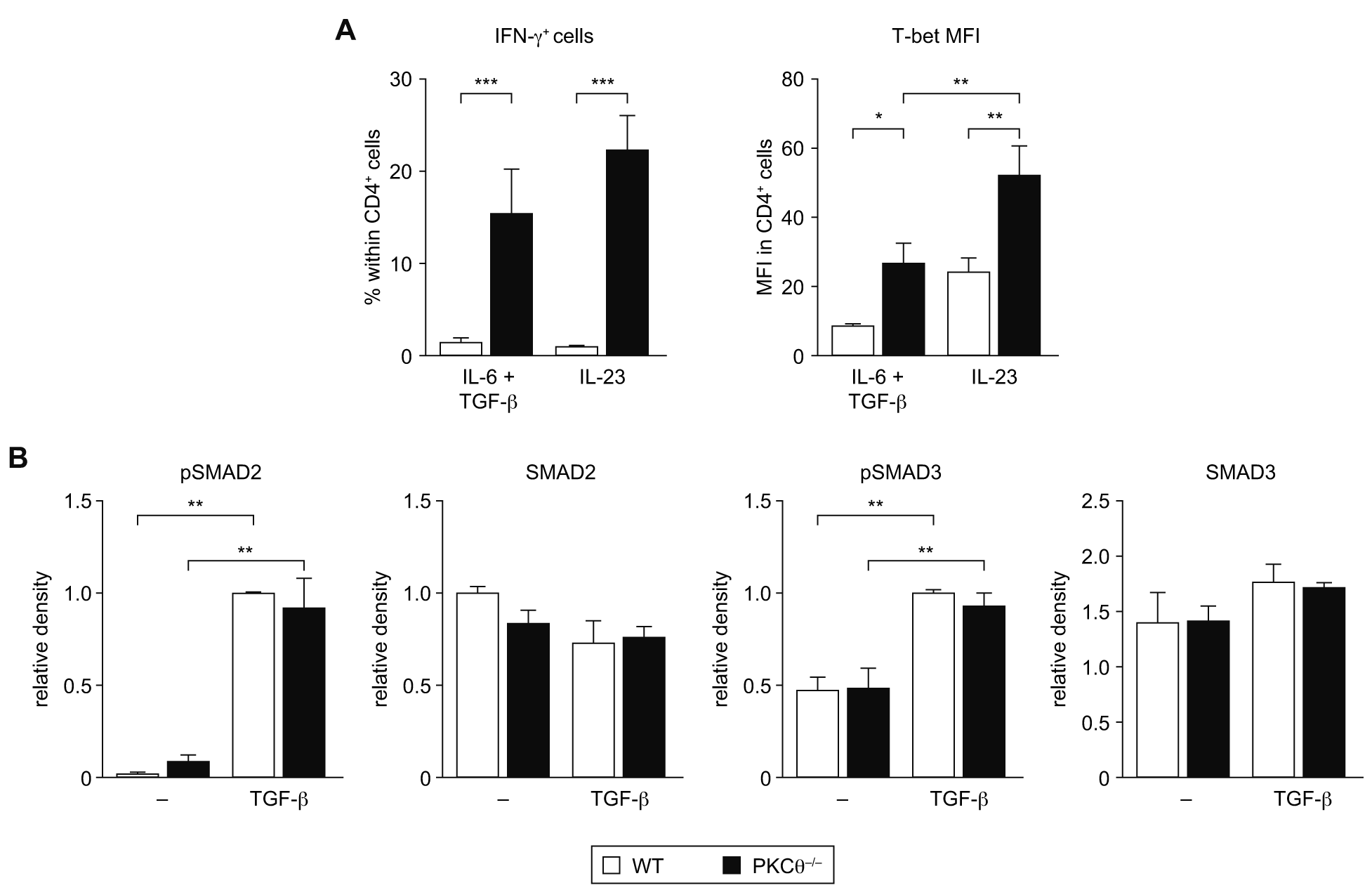

Supplement: Figure S3 — Unaltered responses to IL-23 and TGF-β in PKCθ−/− CD4+ T cells. A) Naïve CD4+ T cells were differentiated under Th17-promoting conditions for 4 days and then re-stimulated in the presence of anti-CD3/CD28 antibodies, with IL-23 or with a combination of IL-6/TGF-β. After 3 days of re-stimulation, the cells were analyzed by intracellular flow cytometric staining for IFN-γ and T-bet expression. Graphs represent combined data of two independent experiments, each with n = 3 per genotype. B) Equal SMAD2/3 phosphorylation and protein levels in freshly isolated WT and PKCθ−/− CD4+ cells treated for 30 min with 5 ng/ml of TGF-β or unstimulated; quantification of the western blot acquisitions. Graphs show combined data from two independent experiments with total n = 3 per each genotype and condition. All graphs represent mean values and error bars indicate +/− SEM. Statistical significance was assessed by a 2-way ANOVA with a Bonferroni post hoc test. (TIF) [file pone.0096401.s003.tif]

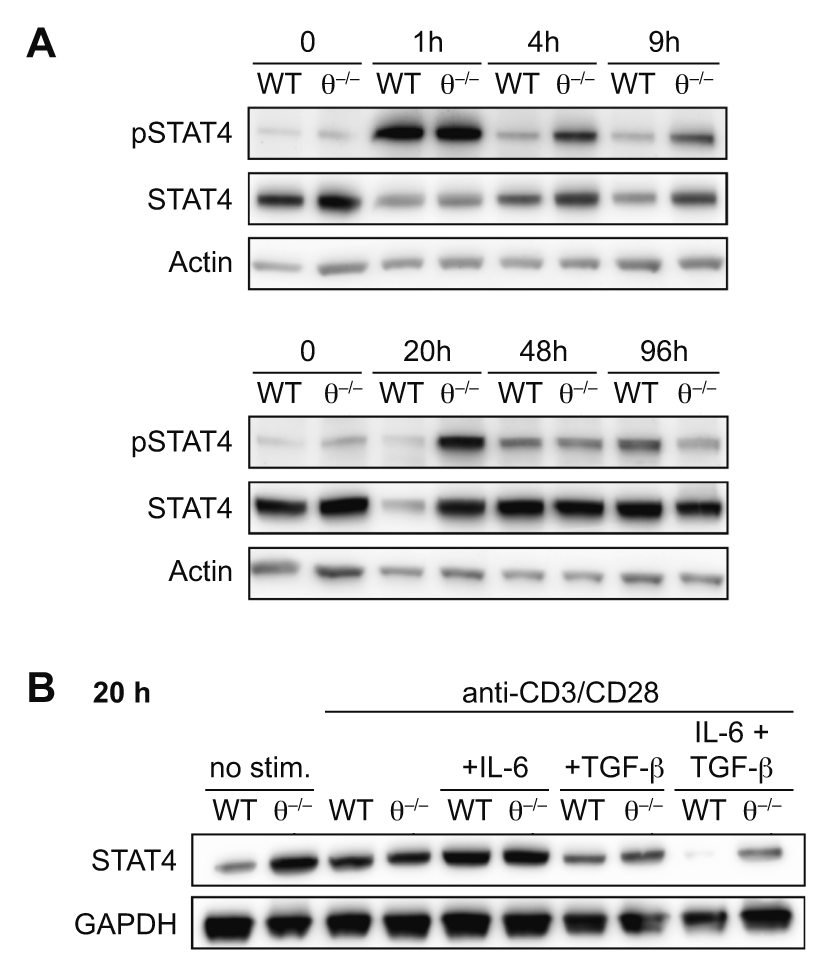

Supplement: Figure S4 — STAT4 regulation during Th17 polarization in WT and PKCθ−/− CD4+ T cells. Naïve WT and PKCθ−/− CD4+ T cells were stimulated by anti-CD3/CD28 antibodies for the indicated times and with the addition of indicated combinations of cytokines. A) Changes of STAT4 phosphorylation and total protein level during Th17 polarization. B) STAT4 regulation in response to different stimulation conditions in the priming phase of Th17 differentiation. Western blots of the representative experiments are shown. (TIF) [file pone.0096401.s004.tif]
